# Microbiome-Specific Statistical Modeling Identifies Interplay Between Gastrointestinal Microbiome and Neurobehavioral Outcomes in Patients With Autism: A Case Control Study

**DOI:** 10.3389/fpsyt.2021.682454

**Published:** 2021-10-20

**Authors:** Minshi Huang, Jun Liu, Kevin Liu, Jierong Chen, Zhen Wei, Zhe Feng, Yuyu Wu, Michelle Fong, Ruiyi Tian, Bryan Wang, Christoph Budjan, Patrick Zhuang, Guobin Wan, Xue-Jun Kong

**Affiliations:** ^1^Affiliated Shenzhen Maternity and Child Healthcare Hospital, Southern Medical University, Shenzhen, China; ^2^Athinoula A. Martinos Center for Biomedical Imaging, Massachusetts General Hospital, Boston, MA, United States; ^3^Harvard Medical School, Boston, MA, United States; ^4^YuNing Clinic, Taipei, Taiwan; ^5^Beth Israel Deaconess Medical Center, Boston, MA, United States

**Keywords:** autism spectrum disorder, gut microbiome, biological pathway, gut-brain axis, multivariable omnibus testing, predictive functional profiling

## Abstract

Autism Spectrum Disorder (ASD) is a neurodevelopmental disorder with unclear mechanisms of pathogenesis. Gastrointestinal microbiome alterations were found to correlate with ASD core symptoms, but its specific role in ASD pathogenesis has not been determined. In this study, we used a case-control strategy that simultaneously compared the ASD gastrointestinal microbiome with that from age-sex matched controls and first-degree relative controls, using a statistical framework accounting for confounders such as age. Enterobacteriaceae (including *Escherichia/Shigella*) and *Phyllobacterium* were significantly enriched in the ASD group, with their relative abundances all following a pattern of ASD > first degree relative control > healthy control, consistent with our hypothesis of living environment and shared microbial and immunological exposures as key drivers of ASD gastrointestinal microbiome dysbiosis. Using multivariable omnibus testing, we identified clinical factors including ADOS scores, dietary habits, and gastrointestinal symptoms that covary with overall microbiome structure within the ASD cohort. A microbiome-specific multivariate modeling approach (MaAsLin2) demonstrated microbial taxa, such as *Lachnoclostridium* and *Tyzzerella*, are significantly associated with ASD core symptoms measured by ADOS. Finally, we identified alterations in predicted biological functions, including tryptophan and tyrosine biosynthesis/metabolism potentially relevant to the pathophysiology of the gut-brain-axis. Overall, our results identified gastrointestinal microbiome signature changes in patients with ASD, highlighted associations between gastrointestinal microbiome and clinical characteristics related to the gut-brain axis and identified contributors to the heterogeneity of gastrointestinal microbiome within the ASD population.

## Introduction

Autism Spectrum Disorder (ASD) is a complex neurodevelopmental disorder (1). Although the etiology of ASD is unclear and likely multifactorial, it likely involves an interplay between genetic and environmental factors (2–4). Recent studies suggest that the gastrointestinal microbiome may play an important role in the pathogenesis of inflammation both in the gastrointestinal and systemically and contribute to dysregulation of the “gut brain axis” (5–10). Other studies point to the potential utility of gastrointestinal microbiome features as biomarkers in assisting ASD diagnosis and screening (11).

The clinical presentation of ASD is highly heterogeneous, and current research points to the existence of multiple subtypes, potentially caused by different routes of pathophysiology and each with diverse comorbid psychiatric and medical conditions (e.g., gastrointestinal symptoms, allergies, sleep disorders). This heterogeneity is not addressed by the conventional DSM5-based behavioral diagnostic criteria. Previously, studies have explored microbiome correlation with GI symptoms in ASD (11, 12). Kong et al. found decreased relative abundance of *Bacteroides* and *Roseburia*, as well as increased relative abundance of *Dorea* and *Prevotella* in ASD patients with abdominal pain (11). Most recently, Plaza-Diaz et al. investigated gastrointestinal microbiome in ASD patients with or without mental regression and found microbiome signatures associated with different psychiatric subtypes (13). In particular, Proteobacteria level was increased exclusively in children with ASD who also present with mental regression. However, previous studies have not analyzed the association between ASD microbiome and clinical factors/comorbidities in a systematic fashion that encompass neuropsychiatric evaluation, dietary factors, immunological indices, and gastrointestinal symptoms. Moreover, there has been a recent explosion of literature on the roles of inflammation and immune regulation on ASD pathogenesis and symptoms presentations. Both clinical studies and ASD mouse models demonstrated aberrations in important immune pathways such as interleukin level, chemokine receptor signaling, and TIM-3 signaling (14–16).

Multiple factors contribute to the composition of the gastrointestinal microbiome, including environmental influences (such as home environment, diet, delivery methods, and breast milk or formula feeding during infancy), age, metabolic indices, gastrointestinal inflammatory state, and potentially host genetic background to a smaller degree (17–19). Previous published studies of the gastrointestinal microbiome in ASD used either first degree relatives or healthy age- and sex-matched individuals as controls (20). Studies have demonstrated that there are differences in the composition of gastrointestinal bacteria between patients with ASD and controls. For example, certain intestinal bacteria are observed to be more abundant in individuals with ASD and they may be involved in the pathogenesis of ASD, which include members of the *Clostridium* and *Sutterella* genus. On the other hand, certain probiotic bacteria such as *Bifidobacteria* have been consistently found to exist at a lower abundance in patients with ASD (20). However, inconsistencies in study designs, small sample sizes, together with heterogeneity of the ASD microbiome may be largely responsible for the enormous inconsistency of the reported ASD microbiome signatures in the literature.

In this study, we aimed to assess the ASD microbiome with an optimized study design that addresses these potential confounding factors by adopting a “double control” strategy: we sampled stool microbiome in individuals with ASD as well as in two parallel control groups including patients' own first-degree relative control and age- and sex-matched unrelated healthy controls. We coupled this study design with a microbiome-specific statistical framework, “multivariate analysis by linear model” (MaAsLin2) recently developed by the Huttenhower lab (21), for subsequent analysis. This approach allows us to better identify core microbial signatures and metabolic pathways unique to ASD, after controlling for age, gender and environmental background and adjusting for age. We hypothesize that the disease state, lifestyle factors and living environment are major drivers of the ASD microbiome, and host genetic background may play a minor role. Because age sex matched healthy controls harbor more pronounced environmental and genetic differences to the ASD patients, as compared to a first-degree relative control group, a trend of microbiome perturbations across groups of the pattern (with age adjustment) of (ASD > first-degree relative control > healthy control) or (ASD < first-degree relative control < healthy control) would provide greater confidence for identifying microbiome biomarkers specific to ASD.

A major knowledge gap in the field is identifying drivers of symptom heterogeneity in patients with ASD, and it is unknown to what extent core behavioral symptoms correlate with, predict, or are predicted by the gastrointestinal microbiome. This information will be crucial in developing strategies for identifying subgroups of patients who may have dysregulation of the gut-brain axis, and who may respond to therapies targeting the gut brain axis. As a secondary goal of the study, we explored microbiome heterogeneity within the ASD group using clinical indices (including GI, dietary habits, immunological functions, and birth history) and behavioral assessments (e.g., ADOS and SRS) using multivariable omnibus testing. We also performed subtype discovery within the ASD microbiome, including clinical factors and behavioral assessments. Focusing on this within-group heterogeneity may offer an alternative path to search for candidate microbes or microbial metabolic pathways that have pathogenic implications for the autism core symptoms and underlying “gut-brain axis” pathophysiology.

## Materials and Methods

### Ethics and Consent

The Internal Review Board (IRB) and Ethical Approval were issued from Shenzhen Maternity and Child Healthcare Hospital. Written informed consent was obtained either from adult subjects who were competent to provide consent or from the parents or legal guardians of children and adults with cognitive impairment. Assent was obtained from subjects who were unable to give consent.

### Participants

#### Inclusion Criteria

##### ASD Group

- Meets the diagnostic criteria in DSM-5 for ASD by two pediatric psychiatrists;
- Aged 3–6 years old;
- Able to complete the autism assessment as scheduled.

##### Healthy Age Sex Matched Control Group

- Typically developing children aged 3–6 years matched with the same gender and age as the ASD group (the age difference does not exceed 3 months).

##### First Degree Family Member Control Group

- Mother or sibling of enrolled ASD children.

Although we intended to enroll siblings as well as mothers, we were only able to recruit mothers as controls due to the paucity of siblings in our study population.

#### Exclusion Criteria

- Having congenital genetic diseases;
- Use of either antibiotics or probiotics within 1 month prior to the study;
- Taking neurological drugs;
- Receiving other dietary supplements.

### Basic Information Questionnaire (Developed by Research Team and Approved by IRB)

Included demographic data of the research subject, history of antibiotic use, intestinal health, oral health, etc.

### ASD-Related Clinical Assessment Tools

#### Gesell Development Diagnosis Scales

This is suitable for the development assessment of children aged 0–6 (22). The assessment is divided into five subcategories: adaptation, gross movement, fine movement, language, and personal-social functions. The scores were omitted from univariate analysis due to missing values.

#### Adaptive Behavior Assessment System-Second Edition

ABAS-II provides a complete assessment of adaptive skills, and behavior rating scale typically completed by parent or primary caregiver (23). It is scored for the 10 Skill Areas—norm-referenced scaled scores and test-age equivalents. For the 3 Adaptive Domains and the General Adaptive Composite (GAC)–norm-referenced standard scores and age-based percentile ranks. In addition, all scores can be categorized descriptively. The scores were omitted from univariate analysis due to missing values.

#### The Childhood Autism Rating Scale

CARS is developed by E. Schopler and others and helps to identify children with autism and determine symptom severity through quantifiable ratings based on direct observation for children over 2 years of age (24). The 15-item rating scales are completed by the clinician, and each item is graded on a 1–4 scale. The total score ranges from 15 to 60; a score of below 30 is considered non-autism, a score of 30–36.5 is considered mild to moderate autism, and a score of 37 or more is considered severe autism based on CARS scale.

#### The Autism Diagnostic Observation Generic

ADOS is a semi-structured standardized assessment of social interactions, language and communication, repetitive, restricted patterns of behavior and interest, and play and imagination. There are four modules (25, 26). The evaluator selected the appropriate module based on the age and language development of the subject and administered it based on the standard protocol. The assessment takes about 40 min for each subject and the cores are recorded on each authorized Chinese version booklet from WPS (27). Raw scores from different ages are converted to standard scores based on the diagnostic algorithm, and the patient's scores are then compared. Calibrated severity scores are converted via standard formula. The evaluators are well-trained and professionally certified in the operation.

#### Chinese Version of the Repetitive Behavior Scale-Revised

The RBS-R is a 44-item self-report questionnaire that is used to measure the breadth of repetitive behavior for ASD individuals (28). It consists of 6 sub-categories: stereotyped behavior (6 items), self-injurious behavior (8 items), compulsive behavior (8 items), routine behavior (6 items), sameness behavior (11 items), and restricted behavior (4 items) for a total score of 43 items. Behaviors are rated on a 4-point scale: 0-Behavior does not occur, 1-Behavior occurs and is a mild problem, 2-Behavior occurs and is a moderate problem, 3-Behavior occurs and is a severe problem. Higher scores indicate more severe repetitive stereotypes. Reliability and validity of the Chinese version of the RBS-R was applicable to 2–7 years old and tested by Li and Jiang in 2013 (29).

#### Social Responsiveness Scale

SRS is a 15-min questionnaire that measures the severity of autistic social impairment from non-existent to severe across the entire range of the autism spectrum (30). It is suitable for children 4–18 years old, with a total of 65 items. It is analyzed from five dimensions including social perception, social cognition, social communication, social motivation, and autistic behavior. Each item has a rating of 0–3: 0 for “No,” 1 for “Sometimes,” 2 for “Often,” and 3 for “Always.” The total score is between 0 and 195 points. The higher the scale score, the more severe the obstacle.

### Intestinal Microbiome Sampling

#### Sample Collection Method

The stool samples were collected by the parents of the subjects following the stool collection kit manufacturers' protocol using the protocol described in Kong et al. 2019 (11). Sample were stored in room temperature for up to 2 days by patients and stored in −80°C freezer in the hospital before shipping to the Beijing Boao Medical Laboratory for processing.

#### Sample Collection Process

##### Autism Subject and Family Member Group

Eligible research subjects first signed informed consent and completed relevant questionnaires. Researchers extracted the results of ASD-related evaluations. The researchers distributed a well-labeled stool sample collection tube [room temperature stool collection kit from Precidiag INC (11)] and instructed the parents on sample collection, date labeling, and sample characteristics recording at home. Samples were kept at room temperature and sent them back to the hospital within 2 days after collection. Samples collected back to the hospital were stored in −80°C freezer before shipping to the Beijing Boao Medical Laboratory for processing.

##### Healthy Control Group

After the sample collection of the ASD group was completed, healthy control group subjects were recruited in a kindergarten in Shenzhen, and the recruitment targets were filtered based on the inclusion and exclusion criteria. Researchers went to the kindergarten for parents to sign the informed consent form, distribute stool collection tubes, instruct teachers and parents to complete sample collection.

#### DNA Extraction and Amplicon Sequencing

Method for DNA extraction from stool is modified from magnetic beads method for soil and stool DNA extraction kit (TIANGEN Biotech, DP712). Following the manufacturer's standard operation procedure, 200 mg stool sample was added into 500 μL buffer solution SA (TIANGEN Biotech, DP712), 100 μL buffer solution SC (TIANGEN Biotech, DP712) and 0.2 g zirconia beads (NIKKATO, YTZ-0.2). This was then placed in a Tgrinder H24 tissue-grinding homogenizer (TIANGEN Biotech) and oscillated at the speed of 6 m/s with 5 sessions of 30 s intervals. DNA sample control was set as: DNA volume ≥ 200 ng, OD260/280 = 1.8–2.1, and main band from DNA electrophoresis > 2,000 bp.

V4-V5 segments were amplified with 515f-y/926r primer pair via PCR (98°C for 3 min, followed by 27 cycles at 98°C for 20 s, 55°C for 30 s, and 72°C for 30 s, and a final extension at 72°C for 2 min). PCR products were purified using AMPure XP Beads (Beckman, A63880) and amplified with Illumina P7 and P5 primer *via* PCR (98°C for 30 s, followed by 6 cycles at 98°C for 20 s, 60°C for 30 s, and 72°C for 30 s, and a final extension at 72°C for 2 min). The post PCR products were purified again using AMPure XP Beads (Beckman, A63889). Library DNA was mixed with fluorescent dye (Qubit dsDNA HS Reagent) for quantitative quality control (concentration ≥ 2 ng/μL) using a Qubit 3.0 fluorometer. Library fragment size was detected by 2% agarose gel electrophoresis and the library was qualified with no primer dimer contamination below 100 bp and library main band around 500 bp. The DNA library was pooled and sequenced on Illumina Miseq sequencing platform with PE 300 bp protocol with overlapping reads.

### Bioinformatics Processing of Amplicon Data

Amplicon sequences were then bioinformatically processed through the DADA2 workflow in R which had been wrapped in the reproducible bioBakery workflow with AnADAMA (31, 32). Briefly, the sequences were demultiplexed and DADA2 run with default parameters to denoise, filter, and trim Illumina data. Next, the open-source R-package assessed the reads for sequence error rates and corrected them on a base-by-base basis. Chimera removal was carried out prior to grouping amplicons into amplicon sequence variants (ASVs). Then, phylogenetic trees were constructed after alignment of sequences using Clustal Omega (33). Finally, ASVs were then taxonomically assigned using SILVA and rRNA specific databases (34).

For functional inference, PICRUSt was run (with default parameters) to predict gene family abundances from marker gene surveys by “multiplying” observed abundances with genes inferred by ancestral state reconstruction in a database of reference genomes (35). Once the taxonomic and functional profiles were constructed, the initial report from the bioBakery workflow was used for basic QC, statistics, and visualizations of the microbial profile data.

All raw data from 16s rRNA Illumina amplicon sequencing have been deposited in The National Centre for Biotechnology Information (NCBI) Sequence Read Archive (SRA, PRJNA687773).

### Statistical Analysis

Next, the Harvard T.H. Chan School of Public Health Microbiome Analysis Core tested disease endpoints against the bacterial communities' alpha and beta diversities (InvSimpson and Bray-Curtis dissimilarity, Unweighted and Weighted UniFrac distances, respectively). Alpha diversity was calculated using the built-in estimate diversity function in Phyloseq (29), and differences in diversity were found using an ANOVA test on a linear model. Beta diversities were calculated using the vegan package in R, and significant differences in community composition were tested using an omnibus univariable PERMANOVA test. Next, we incorporated significant and known influences of the microbiome in multivariable models. Patients < 4 years of age were excluded whenever SRS was used in the modeling, because SRS was only validated for patients ≥ 4 years of age.

Per-feature differences in the composition of the gastrointestinal microbiome from children with autism were explored with the MaAsLin2 tool (21), which used mixed linear models to test for statistically significant associations determined by testing each clade in a hierarchical manner after normalization from counts to relative abundances and log transforming these data. Within each independent response/predictor association multiple comparisons over metadata factor levels were adjusted using a Bonferonni correction, and multiple hypothesis tests overall clades and metadata were adjusted to produce a final Benjamini-Hochberg false discovery rate. MaAsLin2 thus identified microbial organisms and predicted functions that reach a statistically significant association with autism-specific phenotypes. Additionally, we explored the correlations between key microbiota abundances and microbial metabolism pathways through the use of a correlation matrix based on Spearman's rank correlation, using a significance cutoff of α = 0.05.

We then ran Random Forest models to identify which features of the microbiome might predict if a sample was sourced from a child with autism or the healthy controls (children of the same age). Additionally, leveraging Random Forest models we asked if any of the features of the microbiome could predict worsening symptoms of autism among the children with autism spectrum disorder. These models were 10-fold validated with 1,000 random trees, run with 80% of the data as a training set and 20% as the testing set and validated on the full set of data.

We leveraged the tool MMUPHIn (Meta-analysis Methods with a Uniform Pipeline for Heterogeneity in microbiome studies) to test for discrete community structures within these microbiome populations. MMUPHin allowed for the analysis of both known discretely clustered and continuously variable population structures within microbial data by leveraging a meta-analytical approach to compare different PCs or distances within the data to identify population-level structural differences in the data. For all analyses other than alpha diversity, feature tables (both taxonomy and metabolic pathways) were filtered requiring a microbial feature to have at least 0.01% relative abundance in at least 10% of all samples.

Microbiome co-abundance network analysis was performed using the SparCC algorithm via MetagenoNets to elucidate ecological interactions between bacterial features (34). Features were filtered using a prevalence cutoff of 0.005 of maximum prevalence and with occurrences in 10% of samples. After feature filtration and normalization, 69 features remained from the initial total 244 features. Taxa abundances were subsequently normalized by Total Sum Scaling (TSS). All plotted network correlations are significant at *P* < 0.01 based on bootstrapping of 500 iterations.

## Results

### A Microbiome Specific Statistical Model Identities Autism-Associated Gastrointestinal Microbiome Signatures

To characterize the gastrointestinal microbiota and relevant clinical indices associated with ASD, we recruited 39 ASD subjects, 36 first-degree relative controls, and 44 age-/sex-matched healthy controls. The demographic characteristics and selected baseline medical conditions and dietary behaviors of the participants are summarized in Supplementary Tables 1, 2, respectively. Overall, the ASD group showed higher trends in GI severity total score, prevalence of food/skin allergy, as well as scores related to restricted eating behavior, although the differences did not reach statistical significance when compared to other groups. The GI severity total score for children with ASD is 1.77 ± 1.72 whereas the GI severity total score for the HC group is 0.84 ± 1.33 (*P* < 0.01). The mother control group had a GI severity total score of 1.46 ± 1.84.

**Figure 1:**
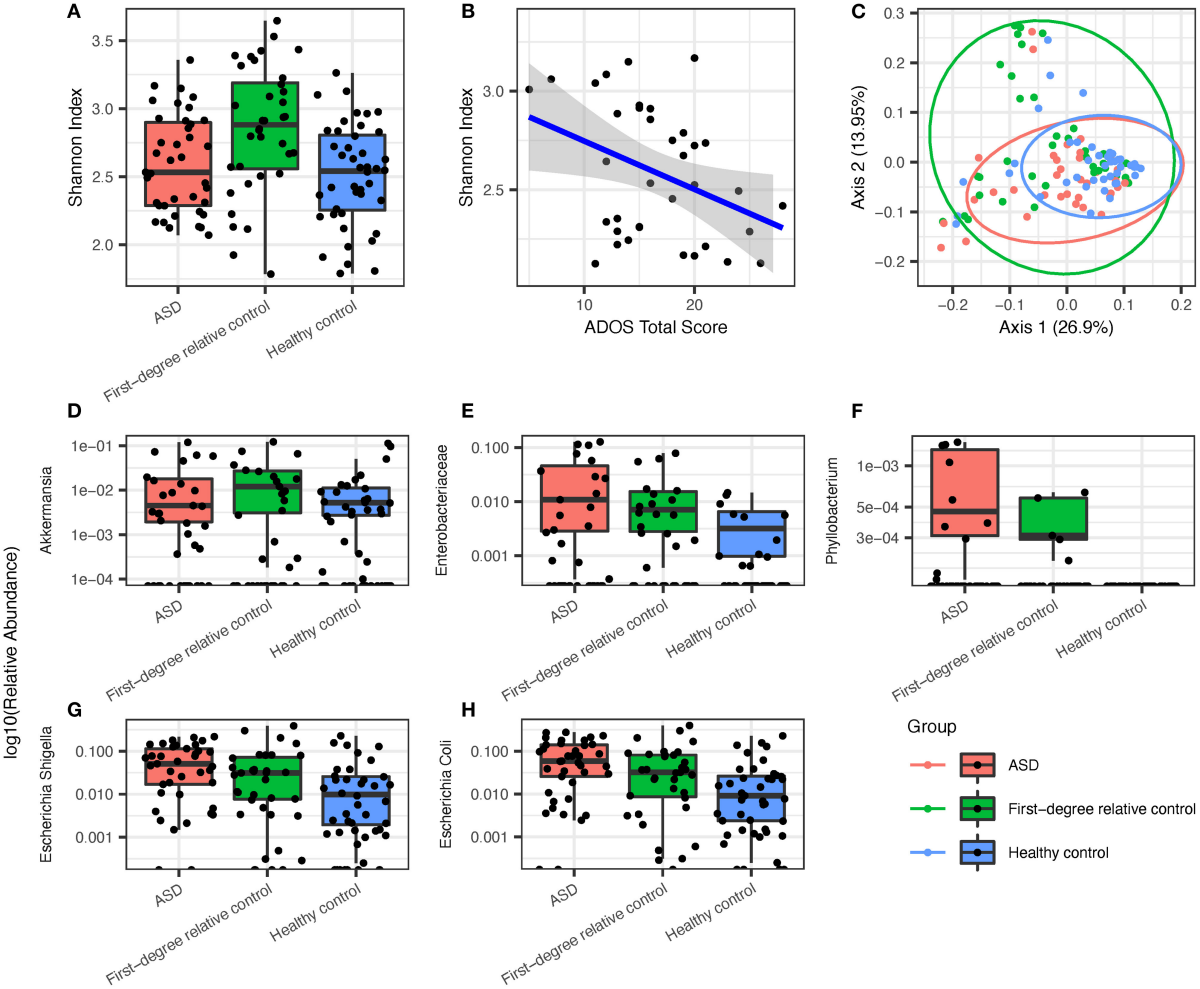
Overview of groupwise subject gut microbiome ecological diversity and differentially enriched gut microbiota that are most prevalent in ASD group subjects, followed by healthy controls and first-degree relative controls, respectively. **(A)** Groupwise comparison of alpha diversity determined via Shannon index in all groups. **(B)** Alpha diversity *via* the Shannon index displays significant inverse correlation with ADOS overall score. **(C)** PCoA of species after filtering (Bray-Curtis) with 95% confidence ellipses. ASD microbiota showed **(D)** a lower abundance of *Akkermansia* but a higher abundance of **(E)** enterobacteriaceae, **(F)** *phyllobacterium*, **(G)** *E. Shigella*, and **(H)** *E. coli* when compared to first-degree relative controls and healthy controls.

Analysis of gastrointestinal microbiome alpha diversity suggested no significant community-level differences between ASD, healthy control, and first-degree family control groups (Shannon index, Figure 1A), although Shannon index showed strong inverse correlation with ADOS total score (Figure 1B, *P* < 0.05, Spearman's ρ = −0.3698). A univariate PERMANOVA analysis of the group-wise differences of beta diversity (Bray-Curtis dissimilarity matrix) showed significant *p*-value and it explained 6% of the variation (*df* = 2, *F-*value = 4.0864, *P* < 0.05). There is a broader distribution of the first-degree relative controls compared to the ASD or healthy group on the principal coordinates analysis (PCoA) plot of beta diversity which most likely reflects the older age and broader age range in the family control group (Figure 1C).

Under the hypothesis that the environmental factors and disease state are the key drivers of microbiome in addition to age, we expect the ASD-specific taxa should show a differential abundance compared to both family member controls and healthy age sex matched control, with a greater difference between ASD and healthy control than between ASD and first-degree relative controls because environmental differences are more pronounced in the healthy control group compared to first-degree relative control group. Using MaAsLin2 mixed linear modeling adjusting for age as a covariate, we found that the relative abundance of *Akkermansia* was significantly lower in ASD compared to first-degree relative controls (Figure 1D, *Q* = 0.04117), and *Phyllobacterium, Enterobacteriaceae*, and *Escherichia/Shigella* are increased in the ASD group as compared to healthy controls (Figures 1E–G, *Q* = 0.1503, 0.06485, and 0.06950, respectively). We next searched for taxa with a pattern of relative abundance that followed a gradient with the highest (or lowest) level seen in the ASD group, intermediate level seen in the first-degree relative control, and lowest (or highest) seen in healthy controls (Figures 1E–G). These taxa included *Phyllobacterium*, Enterobacteriaceae, and *Escherichia/Shigella* (3/4 total number of differentially enriched taxa). The most abundant gastrointestinal species within the Enterobacteriacae family, *Escherichia coli*, also showed a similar trend (Figure 1H).

Next, random forest analysis (Supplementary Figure 1) was used to identify genera important for differentiating between ASD and healthy status. First-degree relative controls were excluded from this analysis because the use of microbiome for disease classification was more relevant for early screening and diagnosis in the age cohort represented by the ASD group and healthy controls. The results showed overlap with taxa identified by mixed linear modeling, with the top discriminatory index, *Escherichia/Shigella*, being also the most significant taxa from MaAsLin2.

### Subjects With ASD Exhibit Alterations in Biological Functions Related to the Gastrointestinal Microbiota and Microbial Network Compared to Controls

**Figure 2:**
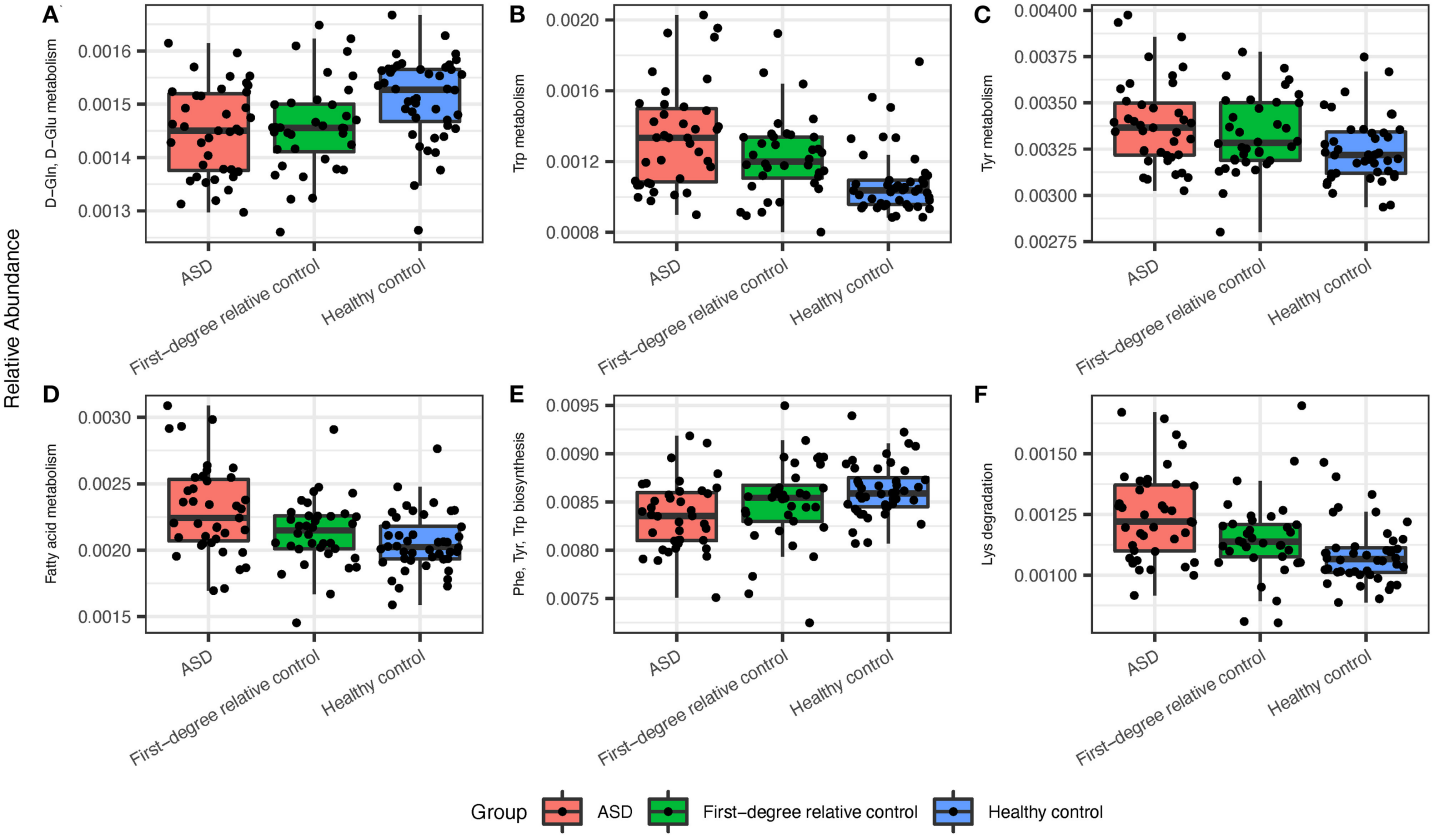
The ASD gut microbiota displayed significant differences in predicted metabolic pathways when compared to healthy controls. **(A)** ASD microbiota expressed lower levels of D-Gln, D-Glu metabolism and higher levels of **(B)** Trp, **(C)** Tyr, and **(D)** fatty acid metabolism. **(E)** ASD microbiota displayed lower levels of Phe, Try, and Trp biosynthesis and **(F)** higher levels of Lys degradation. Significant indices were chosen at a *Q*-value cutoff of 0.1.

Next, we assessed the biological functions (predicted by PICRUSt) associated with the gastrointestinal microbiome by performing groupwise comparisons and visualization. We identified significant differences in microbiome-associated functions between ASD and healthy control groups, while no significant results were found for such indices between ASD and first-degree family controls after adjusting for age (Figure 2). Bacterial metabolism functions that were differentially enriched in patients with ASD include pathways such as Tryptophan metabolism, Tyrosine metabolism, Lysine degradation, and fatty acid synthesis/metabolism. Conversely, tryptophan, phenylalanine and tyrosine biosynthesis (Figure 2; Supplementary Table 3) were deficient in the ASD gastrointestinal microbiome. Interestingly, the age of subjects and the age of the mothers were also strong contributors to differences in some biological functions (Supplementary Figure 2, top 50 enriched or deficient predicted biological functions). Many of these pathways were of pathophysiological relevance to ASD. Next, we performed SparCC network on the genus level within each group of subjects. Patients with ASD demonstrated the densest network of co-occurring genera, compared to first-degree relative controls or healthy controls (Supplementary Figure 3). Furthermore, several differentially abundant microbiota are significantly correlated with bacterial metabolism functions (Supplementary Figure 4).

### GIS Total Score and ADOS Social Affect Scores Are Strongly Predictive of Gastrointestinal Microbiome Alpha Diversity in Patients With ASD

This study is the first to systematically investigate the relationship between gastrointestinal microbiome and ASD clinical presentations. Despite high prevalence of GI symptoms or occult GI issues in ASD, invasive GI investigation remains challenging for this patient population and is frequently under-performed. Sampling of the gastrointestinal microbiome, which only requires stool collection, is relatively simple but financially burdensome. Using an omnibus univariable and multivariable ANOVA test, we asked whether clinical presentations and comorbidities can predict gastrointestinal dysbiosis in ASD patients. The goal was for clinicians to use existing clinical factors to identify the subtypes of ASD patients in whom a greater suspicion of GI abnormality should be raised and followed.

Features that are incorporated into the omnibus modeling were selected from relevant clinical indices, including ADOS and SRS scores (validated clinician assessment and parent assessment for ASD symptoms and functionality, respectively), other behavioral assessments such as CARS and RBS, as well as indices from assessments of GI functions, immune functions, and history of other medical or psychiatric comorbidities. Using the Shannon index as a response variable for alpha diversity, we identified multiple clinical factors, most notably GIS total score, ADOS social affect score, ADOS repetitive and restricted behavior (RRB) score, food refusal, nighttime awakening, and weight, as significant predictors of the Shannon index under a univariate model (*P* < 0.05, Table 1). Next, we attempted to model Shannon index via multiple linear regression and ANOVA to identify predictors based on significant indices from the previous univariate analysis. Predictors included in the multivariate linear model in response to Shannon index include age, weight, GIS total score, “wake up at night,” “refuses food,” ADOS social affect-CSS, ADOS-CSS, and ADOS total score. GIS total score, ADOS SA standard score (CCS) and patient weight were the most significant contributing features (*P* < 0.05, multivariate analysis, Table 1).

**Table 1:** Summary of models and significant univariable model predictors of Shannon alpha diversity with age as a covariate in individuals with ASD of all ages.

**Predictors**	**Univariate *P*-value**	**Multivariate *P-value***
ADOS SA (CSS)	0.015	0.03478^*^
ADOS RRB (CSS)	0.047	NS
ADOS overall (CSS)	0.039	NS
ADOS SA	0.006	NS
ADOS overall	0.008	NS
Weight (kg)	0.027	0.02540^*^
GIS total score	0.043	0.04226^*^
Wake up at night	0.003	NS
Refuses food	0.024	NS

* *P < 0.05*.

### Characterization of Gastrointestinal Microbiota Biomarkers Associated With ASD Core Symptoms

**Figure 3:**
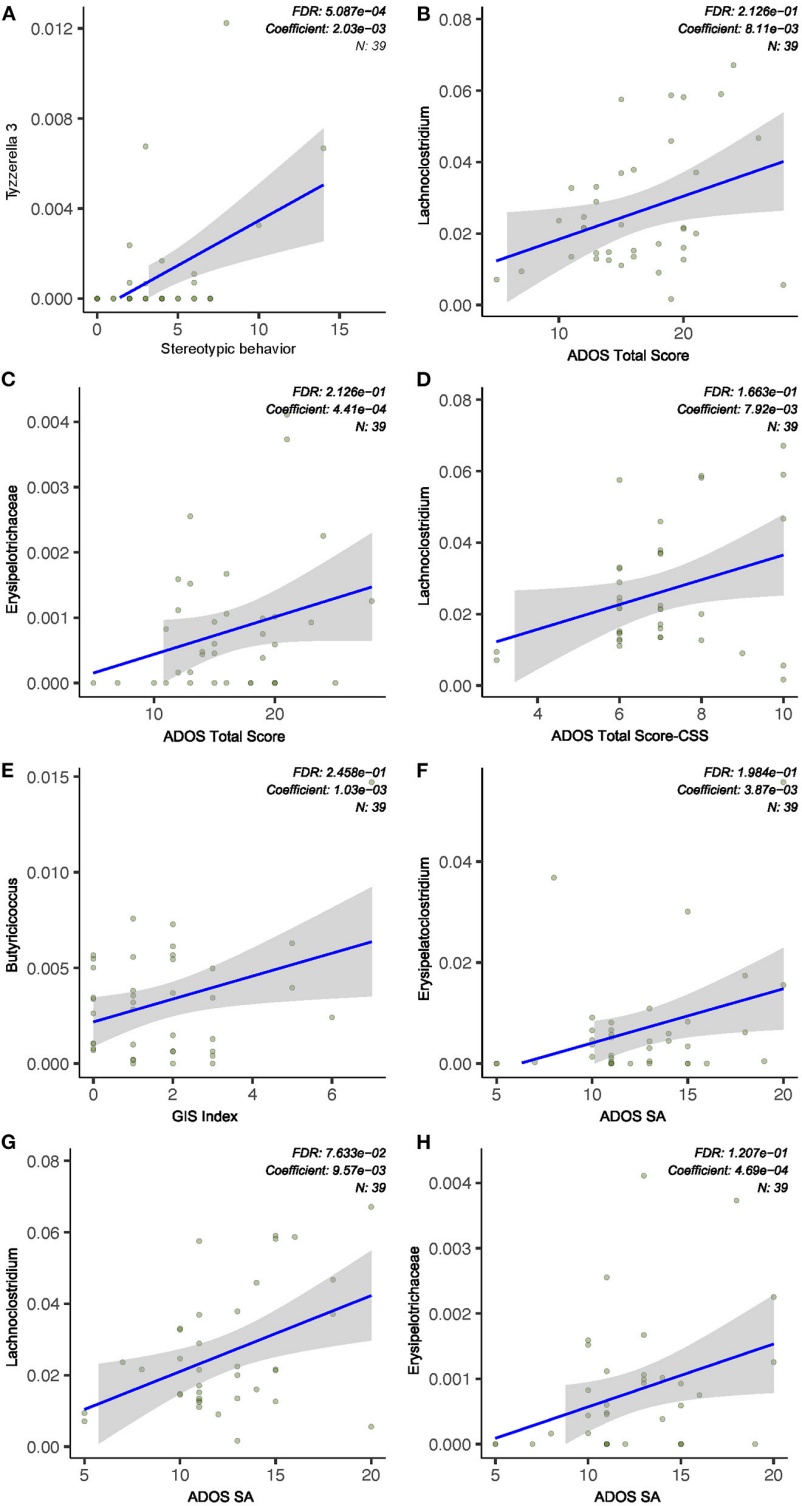
ASD severity was associated with gut microbiota abundance. **(A)** Stereotypic behavior was positively correlated to *Tyzzerella* relative abundance. **(B–D,F–H)** ADOS subscale scores (social affect, SA), total scores, and standard score (CSS) were correlated with relative abundance of bacterial taxa. **(E)** GI severity index score was positively associated with *Butyricicoccus* abundance. Indices were chosen at a univariate *Q-*value (FDR-adjusted *P-*value) cutoff of 0.1.

In an attempt to identify specific gastrointestinal microbial taxa that may independently contribute to the severity of ASD core symptoms, we explored associations between individual bacterial taxa and ADOS scores using linear models. Mixed linear modeling (MaAsLin2) was used to determine the significance of taxa associated with specific clinical variables of interest while adjusting and accounting for other potentially confounding covariates such as age of subjects. The relative abundance of the genus *Erysipelatoclostridium* is positively associated with ADOS social affect (SA) score (*Q* < 0.1, Figure 3). We also found that the genus *Lachnochlostridium* was significantly associated with ADOS SA score, ADOS total score, and ADOS calibrated severity score (CCS, *Q* < 0.1, Figure 3), all of which displayed a direct correlation with the bacterial relative abundance. The only bacterial taxa found to be significantly associated with stereotypical behavior scores is *Tyzzerella* (positive correlation, *Q* < 0.1, Figure 3). Next, we asked if any of the features of the microbiome could predict worsening symptoms of autism among the children with autism spectrum disorder using Random Forest models. Due to the relatively small sample size, the model yielded high specificity but poor sensitivity in both the training (data not shown).

### Clinical Factors Contribute to Gastrointestinal Microbiome Beta Diversity Within the ASD Population

ASD is a highly heterogeneous condition, combining that with highly personalized gastrointestinal communities can make associations difficult. To explore the features contributing to gastrointestinal microbiome diversity within the ASD group, we modeled beta diversity with Bray-Curtis dissimilarity and Weighted/Unweighted UniFrac distance using selected clinical factors (e.g., immune, dietary, and GI conditions), SRS scores (validated for 4 years and above) and ADOS scores. Modeling Bray-Curtis dissimilarity using an omnibus univariable PERMANOVA test showed both intestinal irritability and SRS cognition score are strong predictors in the multivariate model (Table 2, *P* < 0.05). Unweighted UniFrac, which accounts for the phylogenetic relationship between the taxa in generation of dissimilarity matrix, also identified SRS cognition score as significant predictor of beta diversity in a multivariate model, whereas weighted UniFrac identified other gastrointestinal indices, including GIS total score, abdominal pain (a subscale of GIS score), and fatigue (also a subscale of the GIS score), as significant predictors (Table 2, PERMANOVA, *P* < 0.05).

**Table 2:** Summary of omnibus univariate PERMANOVA tests using features from beta diversity metrics and clinical indices in children with ASD aged older than 4 years.

**Clinical index**	**Univariate**	**Univariate**	**Multivariate**	**Multivariate**	**Multivariate**
	***R^**2**^* (%)**	***P*-value**	***F*-statistic (*df* = 1)**	***R^**2**^* (%)**	***P*-value**
**Bray-Curtis**					
CARS overall score	14.512	0.045	1.460	0.043	NS
Intestinal irritability	9.795	0.029	3.459	0.101	0.011^*^
Fatigue	8.143	0.050	1.841	0.054	0.095^.^
SRS cognition (range T)	13.311	0.015	5.388	0.158	0.004^**^
Restricted interests	8.215	0.048	1.1346	0.033	NS
**Unweighted UniFrac**					
GIS total score	7.500	0.033	2.345	0.075	0.011^*^
Constipation	8.149	0.019	1.393	0.045	NS
Abdominal pain	11.257	0.001	3.313	0.106	0.002^**^
Fatigue	7.706	0.025	1.732	0.055	0.064
**Weighted UniFrac**					
CARS overall score	14.830	0.039	0.902	0.032	NS
SRS cognition (range T)	9.131	0.038	2.783	0.099	0.015^*^
Restricted interests	8.013	0.039	1.464	0.052	NS

* *P < 0.05;*

** *P < 0.005*.

## Discussion

In this study, we took advantage of a double-control study design to identify microbial taxa with potential relevance for ASD pathophysiology. We found that the relative abundances of the taxa *Akkermansia, Escherichia/Shigella, Phyllobacterium*, and Enterobacteriaceae were significantly different in patients with ASD as compared to first-degree relative controls or healthy controls. Intriguingly, all the taxa that were enriched in the ASD group, including *Phyllobacterium*, Enterobacteriaceae and *Escherichia/Shigella*, followed a decreasing trend of ASD > first-degree relative control > healthy controls. The fact that the majority of differentially enriched taxa showed such a trend further supports the importance of environmental factors as major drivers in shaping the ASD gastrointestinal microbiome. The Enterobacteriaceae family consists of multiple genera of opportunistic pathogens, with *E. coli* being one of the most abundant members in the stool. *Escherichia/Shigella*, a putative proinflammatory bacteria and a potential opportunistic pathogen, has been previously reported to be enriched in ASD with constipation and positively associated with GI symptoms (36, 37). *Akkermansia*, a genus known to secrete beneficial short chain fatty acids and make positive contributions to the gastrointestinal mucosal health, is deficient in ASD which is consistent with previous studies (38, 39). In addition, the ASD microbiome demonstrates the densest network of co-occurring genera, with the majority of the core co-occurring genera contain species of opportunistic pathogens or are implicated in inflammatory conditions, such as *Tyzzerella, Haemophilus, Veillonella*, and *Enterobactor*. Previous studies have proposed possible mechanisms that altered bacteria can trigger, modify or enhance ASD symptoms, such as through neurotoxic microbial byproducts or intestinal inflammation as a result of certain bacterial species or an altered microbiome (3, 5, 7, 40). However, to causally link the differences in relative abundance to ASD symptoms, further research using animal models are required. From clinical studies and animal models, the importance of inflammation in ASD pathogenesis and disease presentation has been known for a long time. Recent studies have started to unravel the cellular and molecular mechanisms involving the functions of different types of immune cells, ranging from transcriptional factor signaling, cytokine signaling, to pathways involved in cellular proliferation (16, 41, 42). Future studies should investigate whether these mechanisms are involved in the pro-inflammatory processes triggered by ASD-enriched microbe species.

We performed the first comprehensive correlational analysis between ASD microbiome and core clinical symptoms. It is intriguing that only *Tyzzerella* (which belongs to the Lachnospiraceae family and Clostridia class) was found to positively associate with stereotypical behavior. *Tyzzerella* has been associated with a number of human diseases, and a previous study showed a striking enrichment of this taxa in ASD patients with abdominal pain as compared to ASD patients without abdominal pain (12). Multiple studies support the positive relationship between behavioral and GI symptoms in patients with ASD: those with significant GI symptoms tend to exhibit higher levels of repetitive behavior (43). The “motor function hypothesis” states that GI pain and discomfort can be indicative of motor excitation in patients with ASD, while stereotypical behavior can also be a manifestation of abnormal motor function. The strong positive correlation between *Tyzzerella* and both GI pain and restricted repetitive behavior from two independent studies raises the hypothesis that the gastrointestinal microbiome may be a “keystone” link between GI symptoms and stereotypical behavior association. ADOS total score and social affect score are significantly associated with multiple bacterial taxa. For example, the genus *Erysipelatoclostridium* and *Lachnochlostridium* showed a positive correlation with ADOS social affect score and/or total score (*Q* < 0.1). *Erysipelatoclostridium* is a part of normal gastrointestinal microbiota. However, it could become an opportunistic pathogen and it has been identified as a gastrointestinal microbiota biomarker in human patients suffering from *Clostridium difficile* infection and Crohn's disease (44).

ASD patients' gastrointestinal microbiota are highly diverse, which warrants investigation into the key determinants of heterogeneity. This project is the first to systematically explore factors contributing to ASD gastrointestinal microbiome heterogeneity (beta diversity) and alpha diversity. Using a multivariable omnibus modeling framework, we found that SRS cognition score is a significant predictor of beta diversity in the ASD patient cohort using two different beta-diversity indices. This is the first report linking the clinical heterogeneity (as assessed by SRS) with microbiome heterogeneity, in the ASD population. Thus, our results provided further proof-of-principle support of the importance of “gut-brain-axis” in ASD phenotypic presentation. Leveraging machine learning algorithms, we attempted to identify subtypes of ASD based on microbiome and clinical profiles, but due to the relatively small sample size, we did not identify discrete sub-communities in the ASD cohort. Next, by asking whether clinical presentations and comorbidities can predict individual ASD patient's microbiome alpha diversity, we showed a preliminary predictive model of gastrointestinal dysbiosis based on clinical factors for patients with ASD. Our multivariable analysis indicated that ADOS SA standard score, GIS total score (reflecting gastrointestinal discomfort/symptoms), and weight are the strongest predictive factors for alpha diversity (Shannon index). Previous studies have demonstrated association between BMI and alpha diversity (45), but a predictive value of ADOS SA on the alpha diversity has not been reported before. For clinicians, a higher degree of suspicion of GI dysbiosis and occult GI symptoms may be raised in patients who have higher ADOS SA scores and greater degrees of GI discomfort. The clinical utility of this predictive model awaits further validation using a larger sample size and a prospective study design. Overall, this study and others that investigate the underlying pathophysiology or diagnostic biomarkers of the gut brain axis may help to identify subgroups of patients who may benefit from therapies targeting the gut brain axis through modifying the gastrointestinal microbiome (46–48).

Lastly, our study demonstrated predicted abnormalities in stool microbiome-mediated metabolic pathways that are relevant to the functions of the gut-brain-axis in patients with ASD compared to healthy individuals. Our stringent statistical framework took into account patient age and parental age as covariates and identified altered tryptophan and tyrosine metabolism and biosynthesis. The “gut brain axis” is hypothesized as an intricate interplay between the gastrointestinal microbiome, mucosal immune system, enteric nervous system, autonomic nervous system (ANS), and the central nervous system receiving ANS input (40, 49). Recent studies with rodent ASD models suggest that autistic symptoms may be, at least in part, affected by microbial metabolites and their interactions with host immune and neuroendocrine pathways (50, 51). Tyrosine is a precursor to multiple neurotransmitters of the central and enteric nervous system, including dopamine and norepinephrine (52, 53). Consistent with our finding of elevated tyrosine metabolism in the ASD microbiome, previous studies also demonstrated significantly increased Tyrosine metabolism in children with ASD compared to the control group (54). This raises the possibility of altered balance of downstream products such as dopamine and norepinephrine which may exacerbate ASD symptoms or comorbid psychiatric conditions via the ENS/ANS and systemic absorption. In addition, the abundant *E. coli* may be responsible for the predicted elevation in Tryptophan (Trp) metabolism in the ASD stool microbiome which is known to produce many downstream bioactive metabolites (55–57). In addition to the roles of these metabolites in neurotransmitter signaling and inflammation, emerging literature suggests cross talk between metabolism and transcriptional regulation. For example, Tryptophan derivatives have been demonstrated to regulate aryl hydrocarbon receptor (AhR) signaling, which controls cell-specific transcription of important genes in environmental responses and xenobiotic metabolism (58). Future studies are required to further explore the roles of downstream transcriptional signaling with alternations of these key metabolites. Future studies performing stool and serum metabolite profiling are necessary to further explore the metabolic implications of stool microbiome changes.

## Conclusion

This double-controlled study demonstrated that *Escherichia/Shigella*, Enterobacteriaceae, and *Phyllobacterium* are enriched in the ASD group, and their relative abundances all follow a pattern of ASD > first degree relative control > healthy age sex matched control. Furthermore, the microbial biomarkers are significantly associated with ASD core symptoms (measured by ADOS evaluation) and GI symptoms, among other clinical factors. The microbiome-associated biological functions, including Tryptophan and Tyrosine biosynthesis/metabolism were found to be relevant to the pathophysiology of the gut-brain-axis. These findings suggest that some signature changes of microbiome and its associated bacterial metabolic pathways could be of potential diagnostic and subtyping values for ASD. However, future studies are needed to establish mechanisms and causal relationships between the enrichment of pathogenic microbes and ASD pathophysiology with attention to the contribution of microbial metabolites. Larger scaled studies with direct serum and gastrointestinal metabolite sampling would provide further insights. A larger sample is also required to explore the diagnostic and classification utility of the microbial biomarkers identified in our study. The limitations of the current study include: (1) exclusive use of Chinese subjects and the results are not generalizable to other ethnic groups until further studies are performed (2) The relatively high dropout rate and small sample size, which most likely led to the small number of ASD signature taxa identified and underperformance of classification/subtyping by random forest modeling. (3) The use of first-degree relative controls as family member controls, despite accounting for age as a covariate in the statistical analysis. Ideally, future studies can focus on only age-matched sibling controls. (4) use of ADOS-G (Chinese version) instead of ADOS-2.

## Data Availability Statement

The datasets presented in this study can be found in online repositories. The names of the repository/repositories and accession number(s) can be found below: https://www.ncbi.nlm.nih.gov/bioproject/687773, PRJNA687773.

## Ethics Statement

The studies involving human participants were reviewed and approved by Shenzhen Maternity and Child Healthcare Hospital. Written informed consent to participate in this study was provided by the participants' legal guardian/next of kin.

## Author Contributions

X-JK conceptualized the study. X-JK and GW contributed to the project administration and funding acquisition of the study. GW contributed to the provision and organization of study resources. JL and MH contributed to the writing of the original draft. MH and KL contribute to data curation. X-JK, GW, and JL contributed to the methodology and supervision of the study. JL and KL contributed to the formal analysis. KL contributed to the software and validation of the research outcomes. KL, MF, RT, BW, and PZ contributed to the visualization of study outcomes. X-JK, JL, KL, MF, BW, CB, and PZ contributed to the review and editing of the final draft. JC, ZW, ZF, and YW contributed to the investigation and data collection of the study. All authors contributed to the article and approved the submitted version.

## Funding

This study was supported by the Sanming Project of Medicine in Shenzhen (SZSM201512009), Massachusetts General Hospital internal funding (#233263), and Shenzhen Maternity & Child Healthcare Hospital Funding (FYA2017003).

## Conflict of Interest

The authors declare that the research was conducted in the absence of any commercial or financial relationships that could be construed as a potential conflict of interest.

## Publisher's Note

All claims expressed in this article are solely those of the authors and do not necessarily represent those of their affiliated organizations, or those of the publisher, the editors and the reviewers. Any product that may be evaluated in this article, or claim that may be made by its manufacturer, is not guaranteed or endorsed by the publisher.
