# Supplementary material for: Microbiome-Specific Statistical Modeling Identifies Interplay Between Gastrointestinal Microbiome and Neurobehavioral Outcomes in Patients With Autism: A Case Control Study
Source: Front Psychiatry. 2021 Oct 20;12:682454. doi: 10.3389/fpsyt.2021.682454 (PMC8563626; doi:10.3389/fpsyt.2021.682454)
Supplement: Supplementary file 1 [file Data_Sheet_1.docx]

Supplementary Material

# Supplementary Figures and Tables

## Supplementary Figures


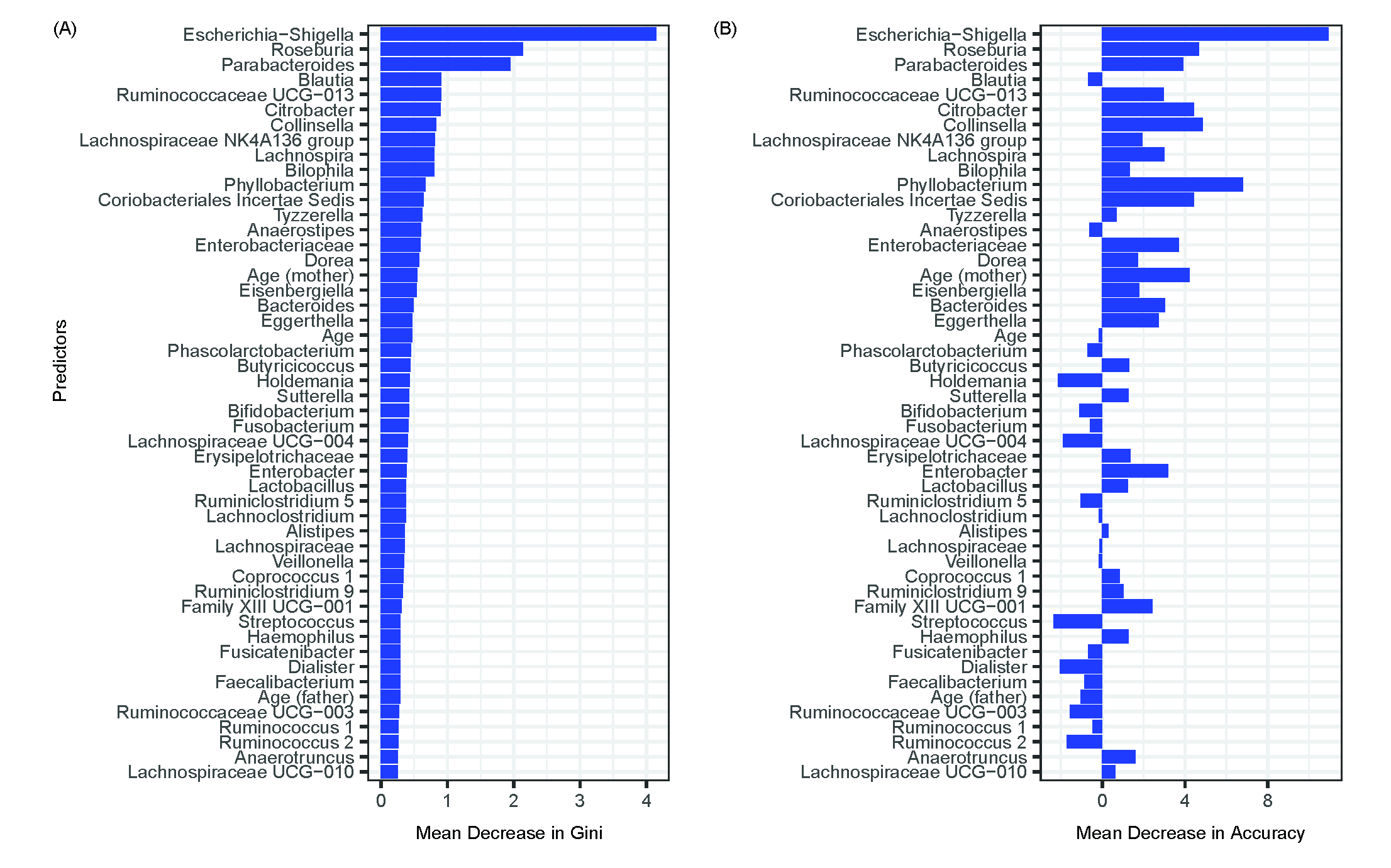


**Supplementary Figure 1.** Random Forest classification of ASD against healthy controls. (A) Features with the highest mean decrease in Gini for ASD classification via random forest. (B) Mean decrease in accuracy associated with random forest classification of ASD subjects (features from panel A).

**
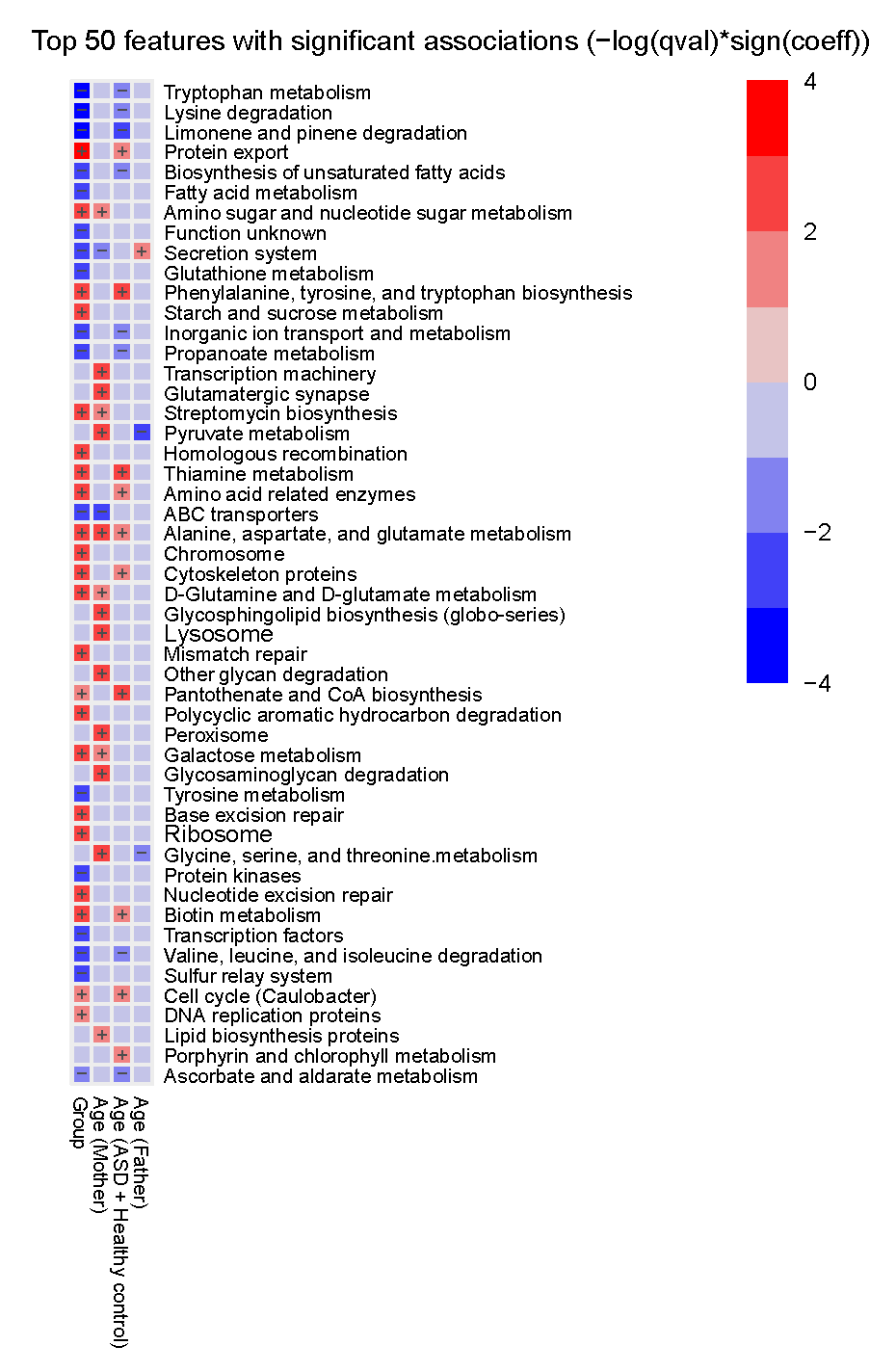
**

**Supplementary Figure 2.** Heatmap of top 50 differentially abundant biological pathways and their association with age compared between the gut microbiome of children with ASD and healthy controls. Top 50 ranks were determined using the formula -log(qval)*sign(coeff).

**
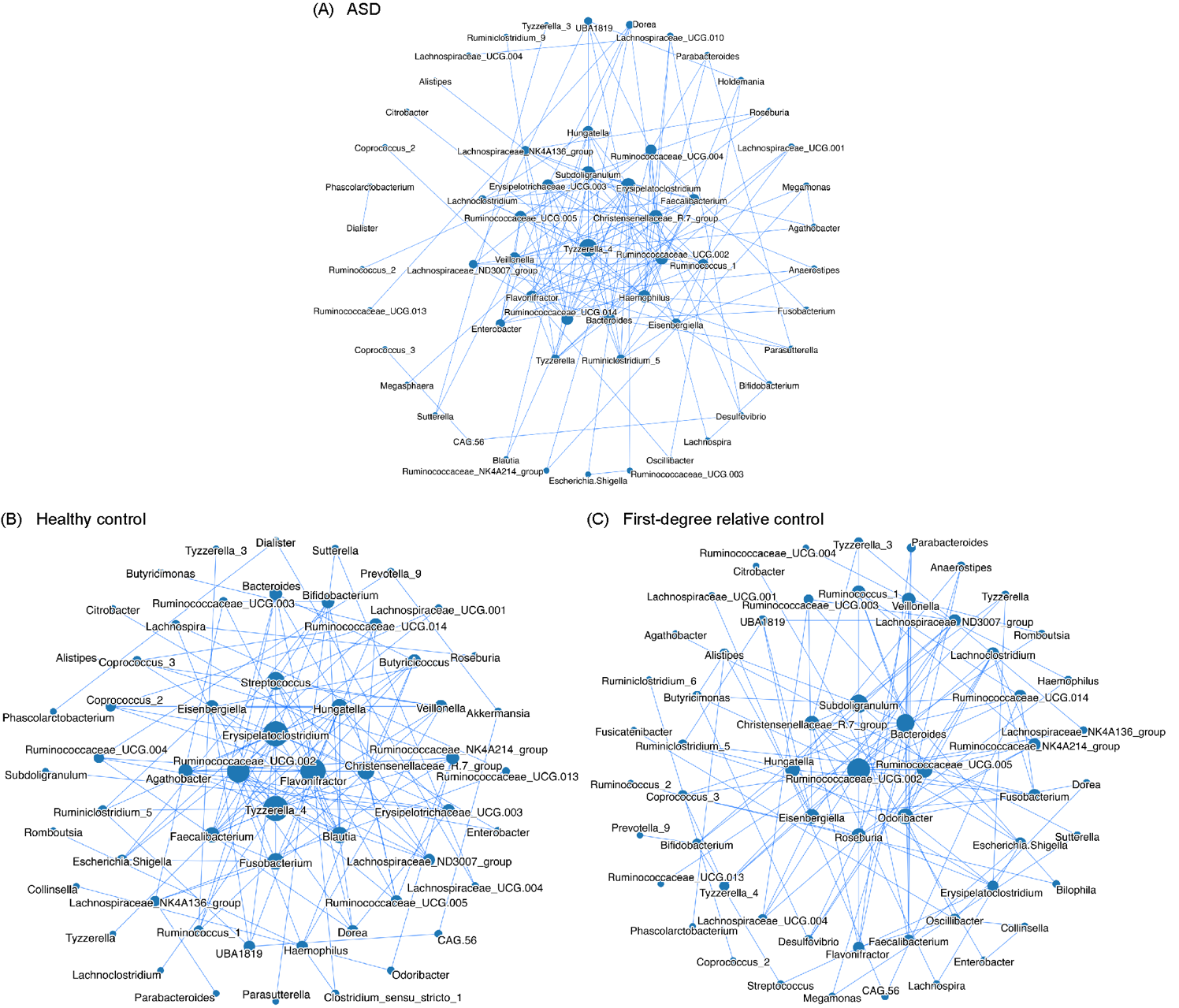
**

**Supplementary Figure 3.** Gut microbiome co-abundance networks. Microbiome co-abundance networks in (A) individuals with ASD, (B) first-degree relative controls, and (C) healthy controls were generated via the SparCC algorithm. Network correlations were filtered with a cutoff of 0.005 of maximum prevalence and with occurrences in 10% of samples and are significant at P < 0.01 based on bootstrapping of 500 iterations.


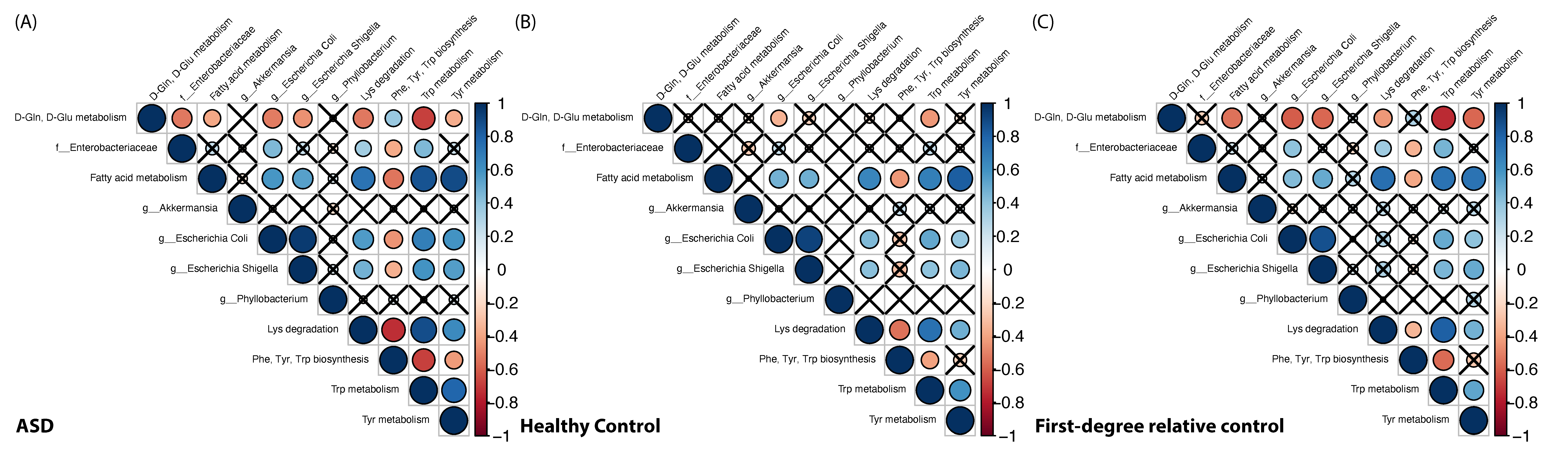


**Supplementary Figure 4.** Spearman’s rank correlation between gut microbiota relative abundance and bacterial metabolic pathways. Non-significant correlations are marked by an “X” based on a significance cutoff of α = 0.05. Correlations shown are for (A) the ASD group, (B) in healthy controls, and (C) in first-degree relative controls, respectively.

## Supplementary Tables

**Supplementary Table 1.** Summary of study participant demographic features.

|  | **ASD**  **(*n* = 39)** | **Healthy control**  **(*n* = 44)** | **First-degree relative control**  **(*n* = 36)** |
| --- | --- | --- | --- |
| Gender |  |  |  |
| Male | 32 | 31 | 0 |
| Female | 7 | 13 | 36 |
| Race | 39 | 44 | 36 |
| East Asian | 39 | 44 | 36 |
| Age (years, mean ± SD) | 4.7 ± 1.1 | 5.1 ± 0.9 | 33.9 ± 5.1 |

**Supplementary Table 2.** Summary of baseline GIS scale and immune-related conditions of study participants (mean ± SD).

|  | **ASD**  **(*n* = 39)** | **Healthy control**  **(*n* = 44)** | **First-degree relative control**  **(*n* = 36)** |
| --- | --- | --- | --- |
| **GIS scale** |  |  |  |
| Total score | 1.6061 ± 1.521 | 0.8571 ± 1.354 | 1.3235 ± 2.0354 |
| *Constipation score* | 0.6364 ± 0.783 | 0.1667 ± 0.3772 | 0.3529 ± 0.485 |
| *Diarrhea score* | 0.000 ± 0.000 | 0.000 ± 0.000 | 0.0588 ± 0.239 |
| *Stool Characteristics score* | 0.0909 ± 0.292 | 0.0714 ± 0.2607 | 0.1176 ± 0.327 |
| *Stool Smell score* | 0.333 ± 0.6455 | 0.1426 ± 0.4174 | 0.3529 ± 0.691 |
| *Gassiness score* | 0.152 ± 0.364 | 0.1426 ± 0.422 | 0.2941 ± 0.579 |
| *GI Pain score* | 0.0303 ± 0.174 | 0.119 ± 0.328 | 0.1176 ± 0.327 |
| *Irritability score* | 0.0606 ± 0.2423 | 0.0238 ± 0.1543 | 0.0294 ± 0.1715 |
| *Wake Up at Night score* | 0.2727 ± 0.626 | 0.1905 ± 0.3974 | 0.2941 ± 0.524 |
| *Abdominal Tenderness score* | 0.0303 ± 0.174 | 0.000 ± 0.000 | 0.000 ± 0.000 |
| *Food Refusal score* | 3.846 ± 1.377 | 3.667 ± 0.8165 | 4.000 ± 0.9979 |
| *Unwilling to Try New Foods score* | 4.038 ± 1.2484 | 3.571 ± 0.9663 | 3.846 ± 0.9811 |
| *Low Food Variety score* | 3.038 ± 1.313 | 2.738 ± 0.9386 | 3.000 ± 0.9979 |
| **Immune-related Conditions** |  |  |  |
| Autoimmune conditions | 1/33 (3.03%) | 1/42 (2.38%) | 3/34 (8.82%) |
| No Autoimmune conditions | 32/33 (96.9%) | 41/42 (97.6%) | 31/34 (91.2%) |
| Allergy to Medications | 1/33 (3.03%) | 0/42 (0%) | 2/34 (5.88%) |
| No Allergy to Medications | 32/33 (96.9%) | 42/42 (100%) | 32/34 (94.1%) |
| Skin Allergy | 6/33 (18.2%) | 5/42 (11.9%) | 6/34 (17.6%) |
| No Skin Allergy | 27/33 (81.8%) | 37/42 (88.1%) | 28/34 (82.3%) |
| Food Allergy | 6/33 (18.2%) | 5/42 (11.9%) | 5/34 (14.7%) |
| No Food Allergy | 27/33 (81.8%) | 37/42 (88.1%) | 29/34(85.3%) |
| Chronic Fatigue | 4/33 (12.1%) | 3/42 (7.1%) | 5/34 (14.7%) |
| No Chronic Fatigue | 29/33 (87.8%) | 39/42 (92.9%) | 29/34 (85.3%) |

**Supplementary Table 3.** Summary of differentially enriched biological pathways between ASD, healthy, and first-degree relative control subjects.

| **Biological Pathway** | ***Q*-value** |
| --- | --- |
| Tryptophan metabolism | < 0.001 |
| Lysine degradation | 0.00192 |
| Protein export | 0.01076 |
| Fatty acid metabolism | 0.00720 |
| Unsaturated fatty acid biosynthesis | 0.00381 |
| Phe, Tyr, and Trp biosynthesis | 0.03011 |
